# Prominent Hepatic Injury in 56-Week-Old Female Mice with Cyclic Guanosine Monophosphate–Adenosine Monophosphate Synthase (cGAS) Deficient

**DOI:** 10.3390/biology15212021

**Published:** 2026-11-06

**Authors:** Chatsuree Suksamai, Thansita Bhunyakarnjanarat, Kanthaporn Dityen, Suwasin Udomkarnjananun, Dhammika Leshan Wannigama, Wiwat Chancharoenthana, Asada Leelahavanichkul, Patcharee Ritprajak

**Affiliations:** 1 Department of Microbiology, Faculty of Medicine, Chulalongkorn University, Bangkok 10330, Thailandthansitadew@gmail.com (T.B.); kanthaporn.d@chula.ac.th (K.D.); 2 Center of Excellence on Translational Research in Inflammation and Immunology (CETRII), Department of Microbiology, Faculty of Medicine, Chulalongkorn University, Bangkok 10330, Thailand; 3 Division of Nephrology, Department of Medicine, Faculty of Medicine, Chulalongkorn University, Bangkok 10330, Thailand; suwasin.u@gmail.com; 4 Department of Infectious Diseases, Faculty of Medicine, Yamagata University, Yamagata 990-9585, Japan; leshanwannigama@gmail.com; 5 Department of Infectious Diseases and Infection Control, Yamagata Prefectural Central Hospital, Yamagata 990-2292, Japan; 6 Pathogen Hunter’s Research Collaborative Team, Department of Infectious Diseases and Infection Control, Yamagata Prefectural Central Hospital, Yamagata 990-2292, Japan; 7 Graduate School, Yamagata Prefectural University of Health Sciences, Yamagata 990-2212, Japan; 8 Biofilms and Antimicrobial Resistance Consortium of ODA Receiving Countries, The University of Sheffield, Sheffield S10 2TN, UK; 9 Department of Clinical Tropical Medicine, Faculty of Tropical Medicine, Mahidol University, Bangkok 10400, Thailand; wiwat.cha@mahidol.ac.th; 10 Tropical Immunology and Translational Research Unit (TITRU), Department of Clinical Tropical Medicine, Faculty of Tropical Medicine, Mahidol University, Bangkok 10400, Thailand; 11 Center of Excellence in Integrative Immuno-Microbial Biochemistry and Bioresponsive Nanomaterials, Department of Microbiology, Faculty of Dentistry, Chulalongkorn University, Bangkok 10330, Thailand

**Keywords:** cGAS deficient mice, aging, inflammation, macrophages, ischemia

## Abstract

Current preclinical studies evaluating cGAS inhibition as a novel anti-inflammatory strategy rely predominantly on young murine models, leaving the impact of aging largely unexplored. At baseline, aged (56-week-old) female mice, in both WT and *cGAS*^-/-^ mice, demonstrated more pronounced oxidative stress (malondialdehyde) and diminished antioxidant capacity (glutathione) relative to young (8-week-old) controls. Upon the induction of liver ischemia–reperfusion injury (IRI), the most severe hepatic damage was provoked exclusively in aged *cGAS*^-/-^ mice. This exacerbated injury was confirmed by elevated liver enzymes, higher histological scores, widespread apoptosis (activated caspase-3), enhanced macrophage accumulation (F4/80^+^ cells), and increased serum cell-free DNA as a tissue damage marker. Additionally, aged *cGAS*^-/-^ mice exhibited a significant surge in serum IL-6 and gut barrier dysfunction (FITC-dextran assay) at 6 h post-injury. Mechanistically, while *cGAS*^-/-^ macrophages showed blunted inflammatory signaling upon lipopolysaccharide (LPS) stimulation, co-incubation with D-galactose, which induces intracellular peroxide accumulation, restored their pro-inflammatory responses to WT levels. Concurrently, starvation protocols revealed that *cGAS*^-/-^ hepatocytes were more vulnerable to mitochondrial oxidative stress (MitoSOX) than WT cells. Ultimately, aging critically modifies the *cGAS*-deficient phenotype, warranting the careful re-evaluation of *cGAS* therapeutic models in aged populations.

## Simple Summary

Ischemia–reperfusion injury (IRI) induced severe liver damage exclusively in aged (56-week-old) *cGAS*-deficient (*cGAS*^-^^/-^) mice, distinguishing them from aged wild-type (WT) and young (8-week-old) mice. Mechanistically, *cGAS*^-/-^ hepatocytes were more vulnerable to starvation-induced mitochondrial injury than their WT counterparts, and oxidative stress heightened the pro-inflammatory capacity of *cGAS*^-/-^ macrophages. Therefore, the influence of aging is a critical variable that should be accounted for before extrapolating results from young subjects.

## 1. Introduction

The liver is an important organ that performs hundreds of essential functions (e.g., nutrient processing and storage, immunity, and protein synthesis), and liver ischemia has a significant impact on the body. Liver ischemia–reperfusion injury (liver IRI) is a critical challenge in several clinical conditions, including liver transplantation, hepatic resection, trauma, and hypoperfusion (shock liver) [1]. The ischemic process reduces oxygen, nutrients, and cellular ATP, shifting metabolism into anaerobic pathways with intracellular acidosis, calcium accumulation, and sinusoidal endothelial cell swelling, while the reperfusion phase exacerbates tissue damage through a massive burst of reactive oxygen species (ROS) and Kupffer cell activation [2]. In an aging society, the impacts of aging on several conditions are significant. As such, aging markedly increases vulnerability to liver injury through a reduction in hepatic blood flow, cellular metabolism, and regenerative capacity, with an elevation in oxidative stress and inflammation [3]. There was a higher incidence of liver injury in the elderly with specific etiologies, for example, hypoperfusion (sepsis) and drug-induced liver injury, due to immunosenescence (age-related decline of the immune system) and the decreased drug clearance capacity [4]. Hence, studies on liver injury in the elderly are specific and need more attention from the scientific community. The aging-induced oxidative stress accumulation in the livers enhances cellular damage and metabolic dysfunction, resulting in an increased vulnerability to secondary injury (e.g., drugs, alcohol, high-fat diets, and ischemia) [5]. Indeed, the aging-induced misfolded protein accumulation causes a loss of mitochondrial repair, leakage of reactive oxygen species (ROS) or free radicals, increased oxidative stress, reduced antioxidants, and cell injury vulnerability [6].

In parallel, cyclic guanosine monophosphate–adenosine monophosphate (GMP-AMP) synthase (cGAS) is a cytosolic sensor recognizing fragmented DNA (microbial or host DNA) in the cytosol to induce cyclic GMP-AMP (cGAMP), the secondary messenger for the downstream activation of the stimulator of interferon genes (STING) [7]. Indeed, both nuclear DNA and mitochondrial DNA (mtDNA) in the cytosol are described as the damage-associated molecular patterns (DAMPs) that induce inflammation in several cell types, including macrophages and hepatocytes [8,9]. Liver ischemia disrupts cellular energy status, induces mitochondrial damage, and increases mtDNA release, escalating inflammatory responses in the liver [10], while cGAS–STING blockade may attenuate liver damage. As such, cGAS^-/-^ mice demonstrate less severe injury in several models than wild-type (WT) mice through the absence of cell stimulation by cytosolic DNA in macrophages [10,11]. In contrast, cGAS^-/-^ mice show more severe inflammation in autoimmune diseases through non-cGAS-dependent pathways [12], highlighting cGAS’s diverse roles across contexts. Mitochondrial damage after liver ischemia may release free mtDNA and nuclear DNA, which cytosolic cGAS recognizes, thereby driving further inflammation in both hepatocytes and macrophages. Loss of cGAS in macrophages limits responses to cytosolic DNA and may reduce M1 pro-inflammatory polarization while enhancing M2 anti-inflammatory polarization [12]. However, cGAS-STING in hepatocytes differs from other cells because STING activity is absent or very low and increases only during chronic inflammation and fibrosis [13]. The loss of cGAS in hepatocytes is associated with an abnormal cell death process (autophagy) [14] based on an enhanced liver injury in young 8-week-old cGAS^-/-^ mice with liver IRI [15]. However, liver injury in aged mice may differ from that in young mice due to several age-related factors [6]. The effects of liver injury in aged mice are of interest.

While several anti-inflammatory cGAS blockages through macrophage interference are being developed [16], some blockages might elevate liver injury due to the unique nature of cGAS-STING in hepatocytes [13]. Despite extensive studies on organ injuries in cGAS^-/-^ mice, the impacts of aging are mostly neglected, with the use of only young mice without a concern for age-related physiologic changes [17]. Manipulating cGAS-STING in both hepatocytes and macrophages might benefit ischemic liver. Although the higher body weight and liver weight in aged mice compared with young mice are major confounding variables that might affect liver injury (possibly more severe injury in the larger livers) [18,19]. This difference is an inherent biological feature of the experimental setting across age groups. Additionally, female mice are frequently preferred in long-term aging studies because group-housed aged males often develop severe, injurious inter-male aggression that requires separation or single-housing, whereas females generally tolerate group housing with fewer social disruptions and welfare complications [20]. As a proof of concept, we performed liver IRI in cGAS^-/-^ female mice at young and aged ages (8 weeks old and 56 weeks old) and conducted several in vitro experiments. We expected different severities of liver damage between WT and cGAS^-/-^ mice across age groups.

## 2. Materials and Methods

### 2.1. Animal and Animal Model

The Institutional Animal Care and Use Committee of the Faculty of Medicine, Chulalongkorn University, Bangkok, Thailand, approved the protocol (IACUC No. 2491034) according to the National Institutes of Health (NIH) criteria. Wild-type (WT) C57BL/6 mice (C57BL/6NJ clone stocked from the Japan/Thailand network) were obtained from Nomura Siam (Bangkok, Thailand), while cGAS^-/-^ mice in a C57BL/6J background were provided by Professor Søren Riis Paludan (Aarhus University, Aarhus, Denmark). All animals were housed in a specific pathogen-free environment in standard clear plastic cages (3–5 mice per cage) with free access to water and food (SmartHeart Rodent; Perfect Companion Pet Care, Bangkok, Thailand) and were subject to light/dark cycles of 12/12 h at 22 ± 2 °C, with 50 ± 10% relative humidity. Thick paper stripes were used for environmental enrichment. Because aged male mice demonstrate territorial aggression and attack other mice in the same cages [21], only 56-week-old female mice were used in the experiments due to an easier husbandry procedure. Although 56-week-old mice are categorized as middle-aged mice (10–15-month-old), partial or early senescent changes are detectable [22] and might be used as an early stage of senescence [23].

A protocol of liver ischemia–reperfusion injury (liver IRI) (70% partial liver ischemia or partial vascular clamping) was followed with some modifications [24,25]. Of note, total hepatic inflow occlusion was avoided because of the elevated risk of mortality and mesenteric congestion-induced intestinal damage, which can disrupt the gut microbiome [26]. Briefly, the mice were anesthetized with isoflurane (Piramal Critical Care, Bethlehem, PA, USA), an abdominal incision was performed, and the portal triad (hepatic artery, portal vein, and bile duct) was occluded using microvascular clamps for 60 min (pallor of the left and medial liver lobes was identified in all mice) on a temperature-controlled surgical bed (Kent Scientific, Torrington, CT, USA). Reperfusion was initiated by clamp removal to restore hepatic blood flow and tissue color, followed by layered closure of the abdominal wall. All mice survived after partial liver ischemia. Sham surgery involved a laparotomy to identify the portal triad [27]. Baseline (0 h) blood samples were collected via tail vein puncture 3 days prior to surgery, followed by subsequent collections at 3 and 6 h post-operation. Notably, the time-course analyses did not include a sham surgery cohort. Mice were sacrificed at the specific time points post-surgery with cardiac puncture under isoflurane anesthesia; organs were harvested post-mortem, and liver tissue was frozen at −80 °C for protein extraction or fixed in 10% paraformaldehyde for histological analysis.

### 2.2. Mouse Sample Analyses

For oxidative stress, livers were sonicated (High-Intensity Ultrasonic Processor, Newtown, CT, USA) in 500 μL of ice-cold phosphate-buffered saline (PBS) containing protease inhibitor cocktail (I3786) (Sigma-Aldrich, St. Louis, MO, USA), and malondialdehyde (MDA) was measured in the supernatant using the MDA assay (Cayman Chemical, Ann Arbor, MI, USA). Meanwhile, livers were sonicated in 2-(N-morpholino) ethanesulfonic acid (MES) buffer (Sigma-Aldrich) before measuring glutathione (GSH) using GSH assays (Cayman Chemical). Serum aspartate aminotransferase (AST) and alanine transaminase (ALT) were measured by EnzyChrom AST (EAST-100) and ALT assay (EALT-100) (BioAssay, Hayward, CA, USA), respectively, while serum cytokines (TNF-α, IL-6, and IL-10) were measured by ELISA (PeproTech, Oldwick, NJ, USA). Serum cell-free DNA (cfDNA) and endotoxin were measured by the Quant-iT™ PicoGreen Assay (Thermo Fisher Scientific, Waltham, MA, USA) and the HEK-Blue LPS detection kit (InvivoGen, San Diego, CA, USA), respectively. The detection of fluorescein isothiocyanate-dextran (FITC-dextran; a non-absorbable carbohydrate) in serum after an oral administration was used for gut permeability evaluation. Briefly, 20 mL/kg FITC-dextran (4.4 kDa) (Sigma-Aldrich) at 25 mg/mL in PBS or PBS alone was orally administered 3 h before surgery, and blood was collected through tail vein nicking at different time points post-surgery. Notably, due to the slow systemic clearance of FITC-dextran, measurements were taken at a single time point per mouse, utilizing independent cohorts for each interval. Serum FITC-dextran was measured by fluorospectrometry (NanoDrop 3300) (Thermo Fisher Scientific) with the excitation and emission wavelengths at 490 nm and 530 nm, respectively. For liver morphological evaluation, 10% paraformaldehyde-fixed samples were embedded in paraffin and stained with hematoxylin and eosin (H&E) color before the semiquantitative evaluation by two observers with a blinded procedure using the Suzuki grading criteria based on congestion (C), vacuolization (V), and necrosis (N) with the following scores: 0 (none C, V, and N), 1 (minimal C and V with <30% N), 2 (mild C and V with <30% N), 3 (moderate C and V with 30–60% N), 4 (severe C and V with >60% N) [28]. Additionally, cellular apoptosis and macrophage infiltration were evaluated via immunohistochemistry using anti-activated caspase-3 and anti-F4/80 antibodies (Cell Signaling Technology, Beverly, MA, USA). The results were quantified as the apoptotic area and the number of F4/80^+^ cells per high-power field (HPF), respectively.

### 2.3. Fecal Microbiome Analysis

Mice from different cages were used for fecal collection to avoid the impact of coprophagy (a habit of consuming feces from other mice in the same cage), and microbiome analysis was conducted following previous publications [29,30]. Briefly, the fecal samples (0.3 g per mouse) were prepared by a FastPrep-24™ Classic bead beating grinder and lysis system (MP Biomedicals, Santa Ana, CA, USA) with a SPINeasy™ DNA Pro Kit (MP Biomedicals) and a DeNovix QFX Fluorometer (DeNovix, DE, USA) (DNA quality and quantity). The prokaryotic 16S rRNA genes were amplified by a Kinnex^TM^ 16S rRNA kit (Pacbio, CA, USA), 16S RNA amplicons were purified by SMRTbell cleanup beads, and the SMRTbell libraries were evaluated by the QIAxcel Advanced (Qiagen, Hilden, Germany) and DeNovix QFX Fluorometer in the Sequel IIe platform. The raw sequences were segmented and demultiplexed into groups based on the Kinnex barcode sequences with the SMRT Link software (https://www.pacb.com/smrt-link/) (accessed on 1 April 2026) and the HiFi-16S-Workflow (https://github.com/PacificBiosciences/HiFi-16S-workflow/) (accessed on 1 April 2026). The DADA2 pipeline describes microbial diversity and community structures using the unique amplicon sequence variants (ASVs), and the microbial taxa were classified from the Silva version 138.2 reference database. The alpha diversity indices (Chao1 richness and Shannon) were computed using DADA2 software version 1.16.0 and tested with a Kruskal–Wallis test (*p* < 0.05). For the beta diversity, the non-metric multidimensional scaling (NMDS) based on the Bray–Curtis dissimilarity was plotted from Phyloseq data. The Kruskal–Wallis sum-rank test was also used for the linear discriminant analysis effect size (LEfSe) to identify bacterial biomarkers that differed significantly in abundant taxa between sample groups. A permutational multivariate analysis of variance (PERMANOVA) was used to determine the significant beta diversity differences at *p* < 0.05 (PERMANOVA value at 0.021). The nucleic acid sequences were submitted to the open-access NCBI Sequence Read Archive under accession number PRJNA1266403.

### 2.4. In Vitro Experiments

Here, macrophages were tested to support the impacts of oxidative stress, while hepatocytes were used to evaluate the injury from cell starvation. As such, bone marrow-derived macrophages from 8-week-old WT and cGAS^-/-^ mice were isolated as previously described [31]. Briefly, mouse femurs and tibias were centrifuged (6000 rpm at 4 °C) and incubated for seven days with Dulbecco’s Modified Eagle medium (DMEM) and conditioned media from the L929 cell line (containing macrophage colony-stimulating factor) to differentiate pluripotent stem cells into macrophages. Then, macrophages (1 × 10^6^ cells/well) were incubated with DMEM (media control), D-galactose (D-gal) (Sigma-Aldrich) 100 mM, lipopolysaccharide (LPS from *E. coli* 026:B6) (Sigma-Aldrich) 100 ng/mL, or D-gal with LPS for 24 h before the analysis of supernatant cytokines by ELISAs (PeproTech) and expression of several genes from the cells using quantitative real-time polymerase chain reaction (PCR). For PCR, the RNA was extracted from cell pellets using a FavorPrep^TM^ RNA mini kit (Favorgen, Ping Tung, Taiwan), quantified by a NanoDrop OneC Microvolume UV-Vis Spectrophotometer, cDNA was synthesized using a cDNA reverse transcription kit (Thermo Fisher Scientific), and measured by SYBR^®^ Green PCR Master Mix with the Applied Biosystems QuantStudio 6 Flex Real-Time PCR System (Thermo Fisher Scientific). Results are shown as relative quantification using the comparative threshold method (delta-delta Ct; 2−ΔΔCt), normalized to *β-actin* (an endogenous housekeeping gene). The primers for mouse genes were derived from previous publications (Table 1). In parallel, the DNA breaks in macrophages were determined using immunofluorescence following a previous publication [32]. Briefly, the cells were fixed with 4% paraformaldehyde in Tris-buffered saline (TBS; pH 7.6) for 15 min, permeabilized with 0.1% Triton X-100, blocked with 2% bovine serum albumin, incubated overnight at 4 °C with phospho-histone H2A.X (Ser139) rabbit monoclonal antibody (Cell Signaling), and visualized by goat anti-rabbit IgG with Alexa Fluor 488 (green color) (Abcam, Cambridge, UK). The actin filaments and nuclei were labeled with Alexa Fluor 555/568 phalloidin (red color) (Invitrogen™, Carlsbad, CA, USA) and DAPI (Sigma-Aldrich), respectively. Images were visualized using a Lattice SIM 5 super-resolution microscope (Zeiss, Oberkochen, Germany), and fluorescence intensity per cell was quantified using ZEN 3.11 software (Zeiss, Germany) at 630× magnification in 10 randomly selected fields per slide. All fluorescence data were normalized to the untreated control group.

In parallel, hepatocytes were isolated from mice as previously described [33]. Briefly, livers were perfused in situ via portal veins with modified Hank’s Balanced Salt Solution (HBSS) (HyClone™, Cytiva, Marlborough, MA, USA) with Liberase™ (Sigma-Aldrich, St. Louis, MO, USA) to disrupt cell junctions. Then, livers were transferred into cold HBSS, filtered through a 70 μm cell strainer, and centrifuged to separate hepatocytes into the low-glucose Dulbecco’s Modified Eagle Medium (DMEM) with 5% fetal bovine serum (FBS) and 1% penicillin-streptomycin. After that, the cells were resuspended in Percoll™ PLUS density gradient media (Cytiva, Marlborough, MA, USA), centrifuged (200× *g*, 10 min, 4 °C), and seeded onto collagen-coated plates at a density of 5 × 10^5^ cells per well (6-well plates) for 3 h before replacing the media with William’s E medium supplemented with 2 mM glutamine and 1% penicillin-streptomycin. Subsequently, a starvation protocol (glucose-free media) [34] or control medium was incubated with the cells for 24 h before collecting the cells and supernatant. The 2′3′-cGAMP in the supernatant was measured by an ELISA kit (Cayman Chemical), while the expression of *cGAS*, *TNF-α*, and *IL-6* was evaluated by qPCR as mentioned above. To determine mitochondrial injury, the starvation or control hepatocytes were washed with pre-warmed HBSS at 37 °C for 30 min in the dark with 500 nM MitoSOX™ Red (a specific indicator of mitochondrial superoxide) (Invitrogen™) before the quantification of the fluorescence intensity using a Varioskan™ (Thermo Fisher Scientific) Flash microplate reader using the excitation at 396 nm and emission at 610 nm. The relative fluorescent unit (RFU) of MitoSOX was calculated using the following equation: RFU = (measured value) × (number of cells after measurement)/(number of cells before measurement).

### 2.5. Statistical Analysis

Data are expressed as the mean ± standard error of the mean (SEM). Statistical comparisons involving two independent variables (age and genotype) were performed using a two-way analysis of variance (ANOVA) followed by Tukey’s honestly significant difference (HSD) post hoc test. For non-parametric data, such as the Suzuki scores for liver injury, the Kruskal–Wallis test followed by Dunn’s post hoc test was applied. Additionally, two-way ANOVA was utilized to assess the age × genotype interaction for key outcome variables. For the in vitro experiments, the one-way ANOVA followed by Tukey’s analysis was used. All statistical analyses were performed in GraphPad Prism version 10.0 (La Jolla, CA, USA), and *p* < 0.05 was considered statistically significant.

## 3. Results

### 3.1. More Severe Hepatic Cell Death in Aged cGAS^-/-^ Mice, a Possible Impact of Prominent Hepatic Oxidative Stress

**Figure 1:**
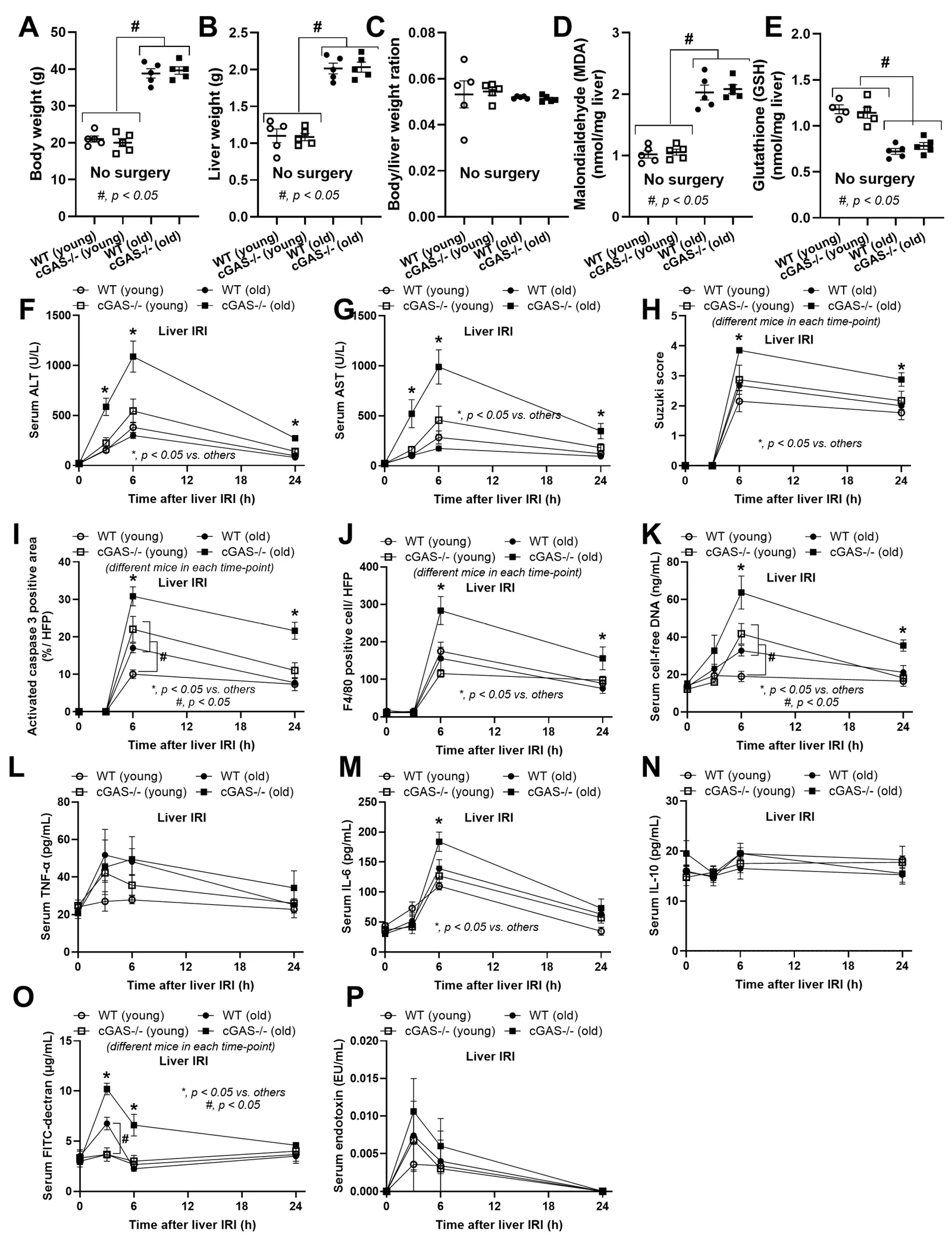
Baseline characteristics of young (8-week-old) and aged (56-week-old) wild-type (WT) and *cGAS*-deficient (*cGAS*^-/-^) mice, as indicated by body weight (**A**), liver weight (**B**), liver/body weight ratio (**C**), hepatic malondialdehyde (MDA) oxidative stress (**D**), and hepatic glutathione (GSH) antioxidant (**E**), are demonstrated. Timepoints of several parameters after liver ischemia–reperfusion injury (liver IRI) in WT and *cGAS*^-/-^, as indicated by liver enzymes, including alanine transaminase (ALT) and aspartate aminotransferase (AST) (**F**,**G**); histological score based on the hematoxylin and eosin color (Suzuki score) (**H**); apoptosis area (activated caspase-3 positive cells) (**I**); abundance of macrophages in livers (F4/80-positive cells) (**J**); serum cell-free DNA (**K**), serum cytokines (**L**–**N**); gut permeability defect (FITC-dextran) (**O**); and endotoxemia (**P**), are demonstrated. *n* = 4–5 per group for no surgery; *n* = 7 per time point in experiments using the same mice; and *n* = 6 per group for 0, 3, and 6 h in experiments with different mice at these time points. *, *p* < 0.05 versus others; #, *p* < 0.05.

**Figure 2:**
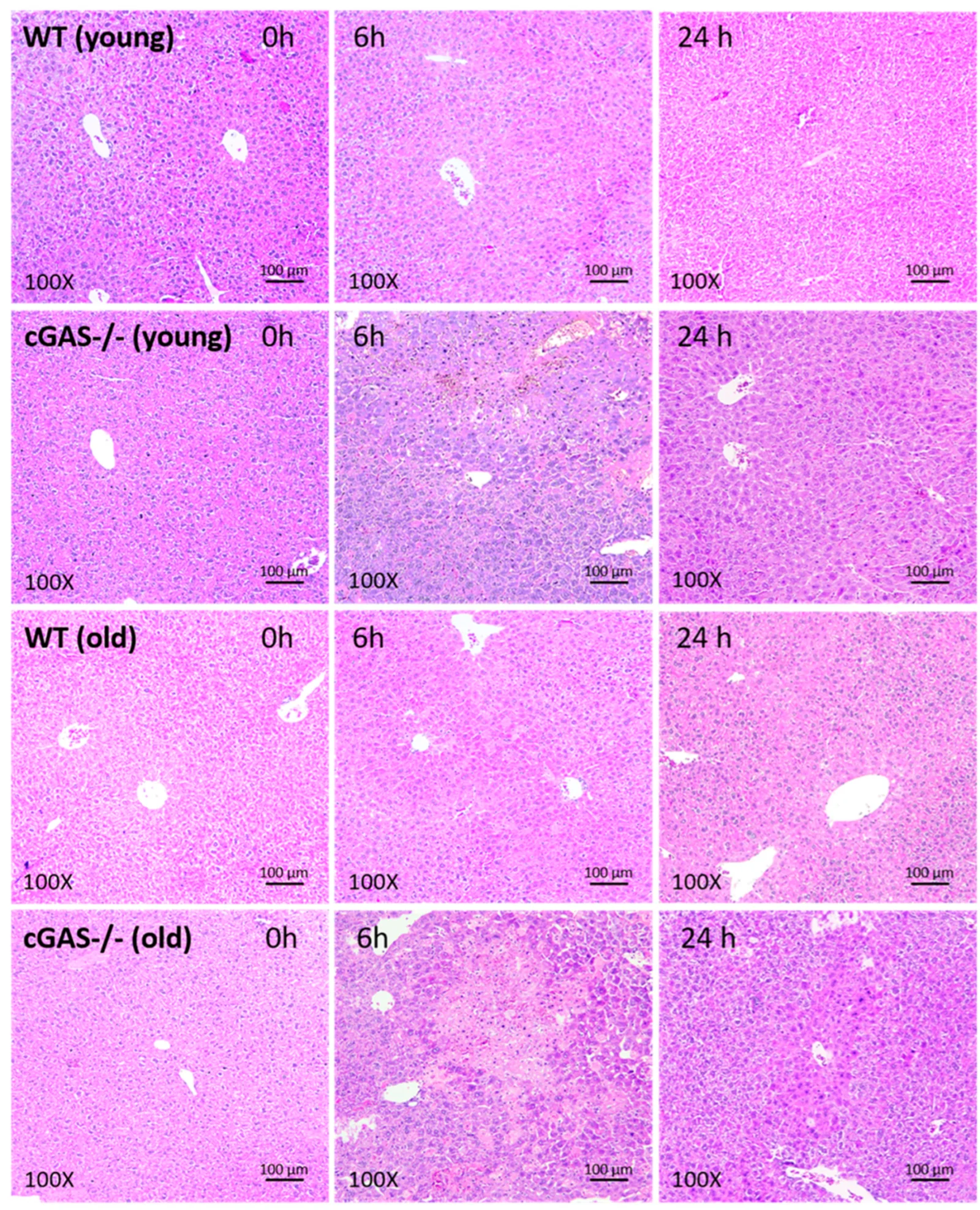
Histological characteristics of liver injury in young (8-week-old) and aged (56-week-old) wild-type (WT) and *cGAS*^-/-^ mice at baseline (0 h) and at 6 and 24 h post-ischemia–reperfusion injury (IRI), as assessed by hematoxylin and eosin (H&E) staining.

**Figure 3:**
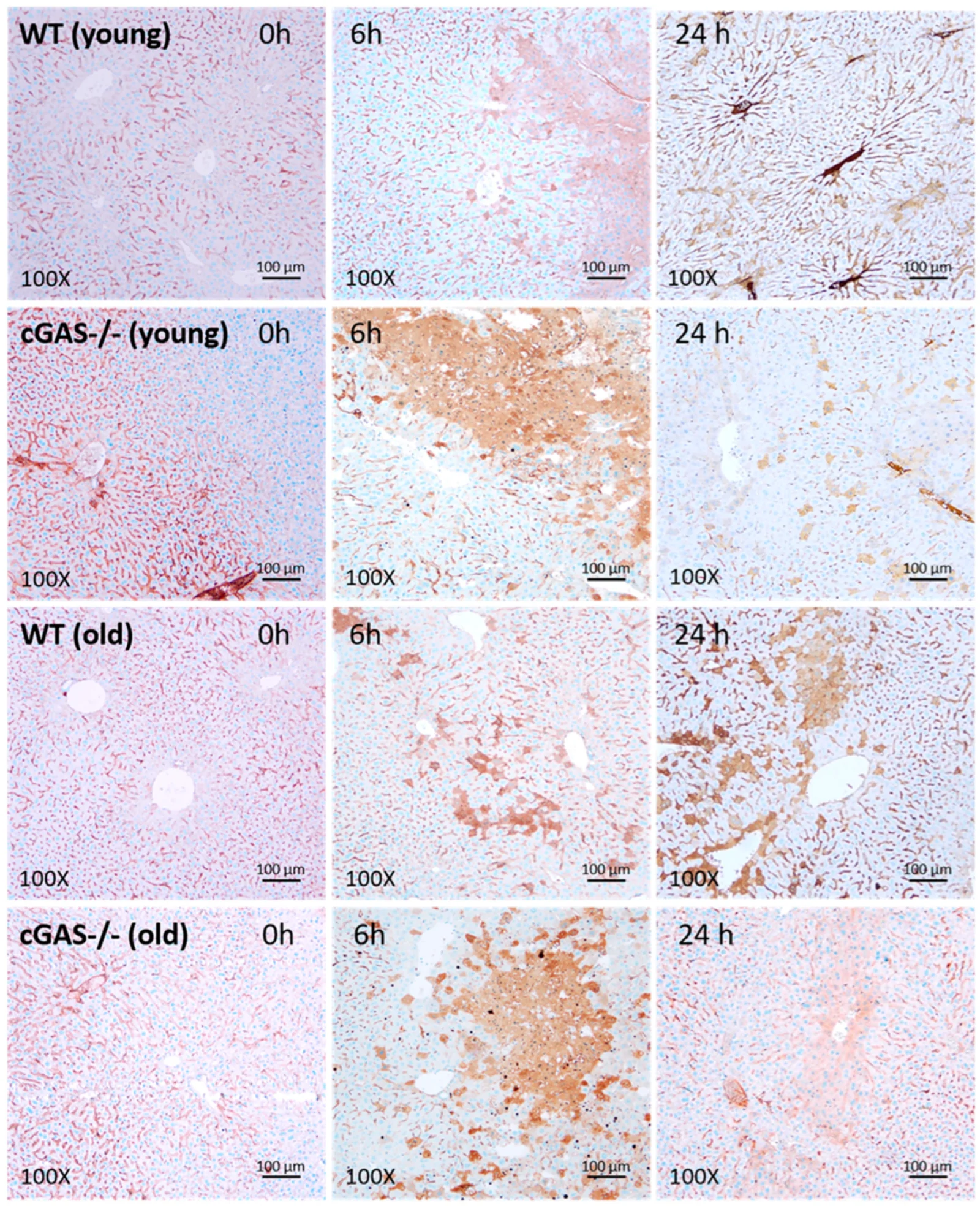
Liver apoptosis in young (8-week-old) and aged (56-week-old) wild-type (WT) and *cGAS*^-/-^ mice at baseline (0 h) and at 6 and 24 h post-ischemia–reperfusion injury (IRI), as assessed by activated caspase-3 immunohistochemical staining.

**Figure 4:**
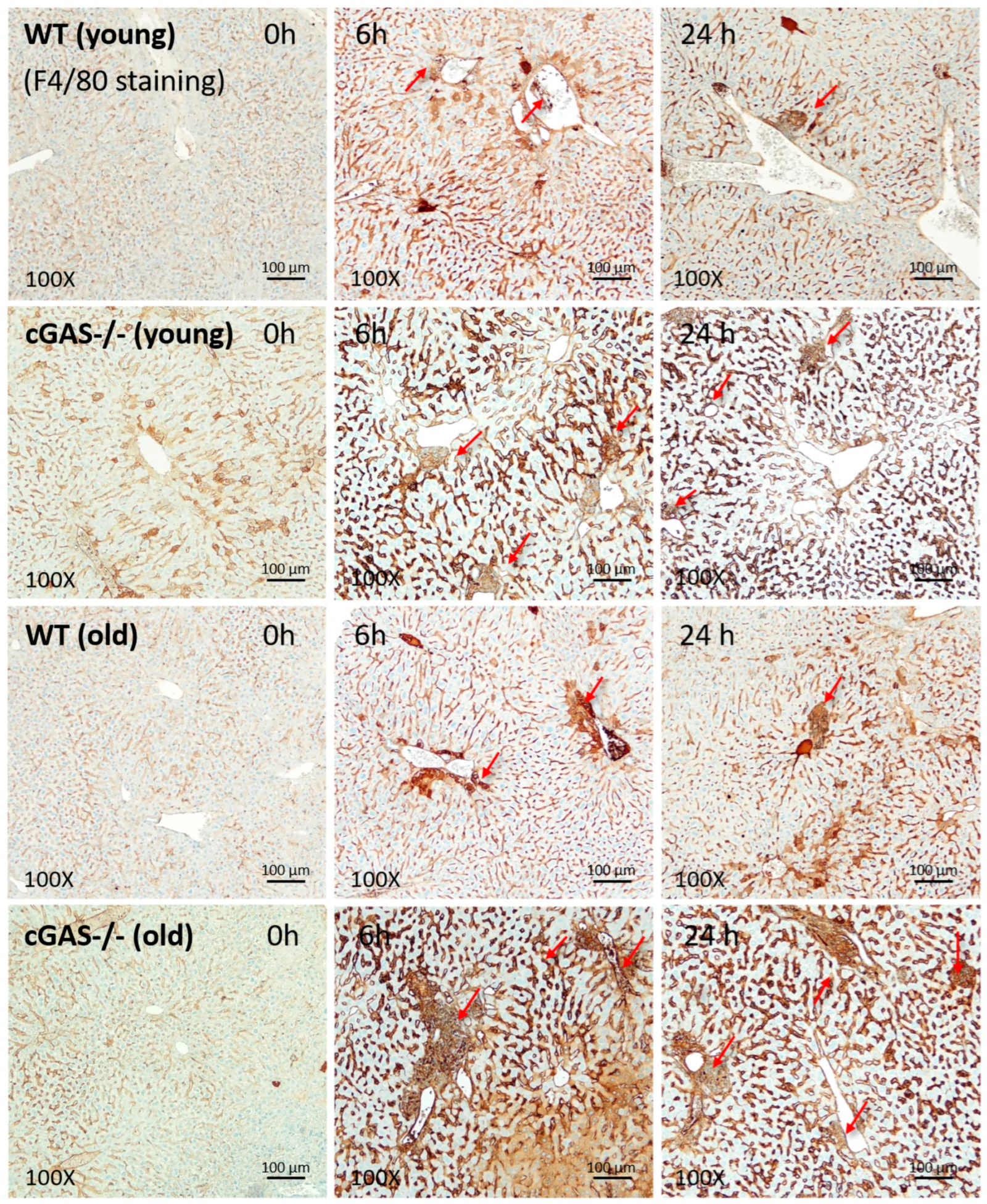
Hepatic macrophage abundance in young (8-week-old) and aged (56-week-old) wild-type (WT) and *cGAS*^-/-^ mice at baseline (0 h) and at 6 and 24 h post-ischemia–reperfusion injury (IRI), as assessed by F4/80 immunohistochemical staining. F4/80-positive cells are indicated by red arrows.

To test the hypothesis, 8-week-old (young mice) and 56-week-old (aged mice) WT and *cGAS*^-/-^ female mice were used. Although 56-week-old mice are technically considered middle-aged, they exhibit early senescent changes [23] and are thus referred to as aged (or older) mice in this study. The aged mice had higher body and liver weights but a similar liver-to-body weight ratio (Figure 1A–C). There was higher oxidative stress (malondialdehyde; MDA) and lower antioxidant (glutathione; GSH) in the livers from aged mice, supporting previous publications [35]. Following liver ischemia–reperfusion injury (IRI), aged *cGAS*^-/-^ mice exhibited exacerbated hepatic injury compared to all other groups. This was evidenced by significantly higher serum alanine transaminase (ALT) and aspartate aminotransferase (AST) levels at 3, 6, and 24 h, whereas no significant differences were observed among the remaining cohorts (Figure 1F,G). In parallel, histological analyses at various time points were performed using independent cohorts of mice. Accordingly, *cGAS*^-/-^ mice exhibited the most severe tissue injury at 6 and 24 h, as assessed by Suzuki’s histological scores, apoptotic area (activated caspase-3 staining), and macrophage infiltration (F4/80-positive cells) (Figure 1H–J, Figure 2, Figure 3 and Figure 4). F4/80 was utilized solely as a marker of macrophage abundance without assessing specific markers of macrophage activation. Meanwhile, the apoptosis area, but not other histological scores, was higher at 6 h in young *cGAS*^-/-^ and aged WT mice than in young WT (Figure 1H–J). The high intensity of liver damage at 6 and 24 h in *cGAS*^-/-^ mice was also supported by the highest serum cell-free DNA (cfDNA) levels (Figure 1K) [36,37]. At 6 h of the liver IRI model, serum cf-DNA in young cGAS^-/-^ and aged WT mice was higher than in young WT (Figure 1K), similar to the 6 h apoptosis area (Figure 1I). At 6 h post-liver IRI, aged *cGAS*^-/-^ mice demonstrated transiently elevated serum IL-6, but not TNF-α and IL-10, and gut permeability defects (FITC-dextran assay), but not serum endotoxin (Figure 1L–P). The *p*-values for the age × genotype interaction, derived from two-way ANOVA for principal outcomes (ALT, AST, histological injury, apoptosis, and macrophage accumulation), are presented in Supplementary Table S1.

### 3.2. Different Liver Injury-Induced Gut Microbiota Between cGAS^-/-^ and WT Aged Mice

**Figure 5:**
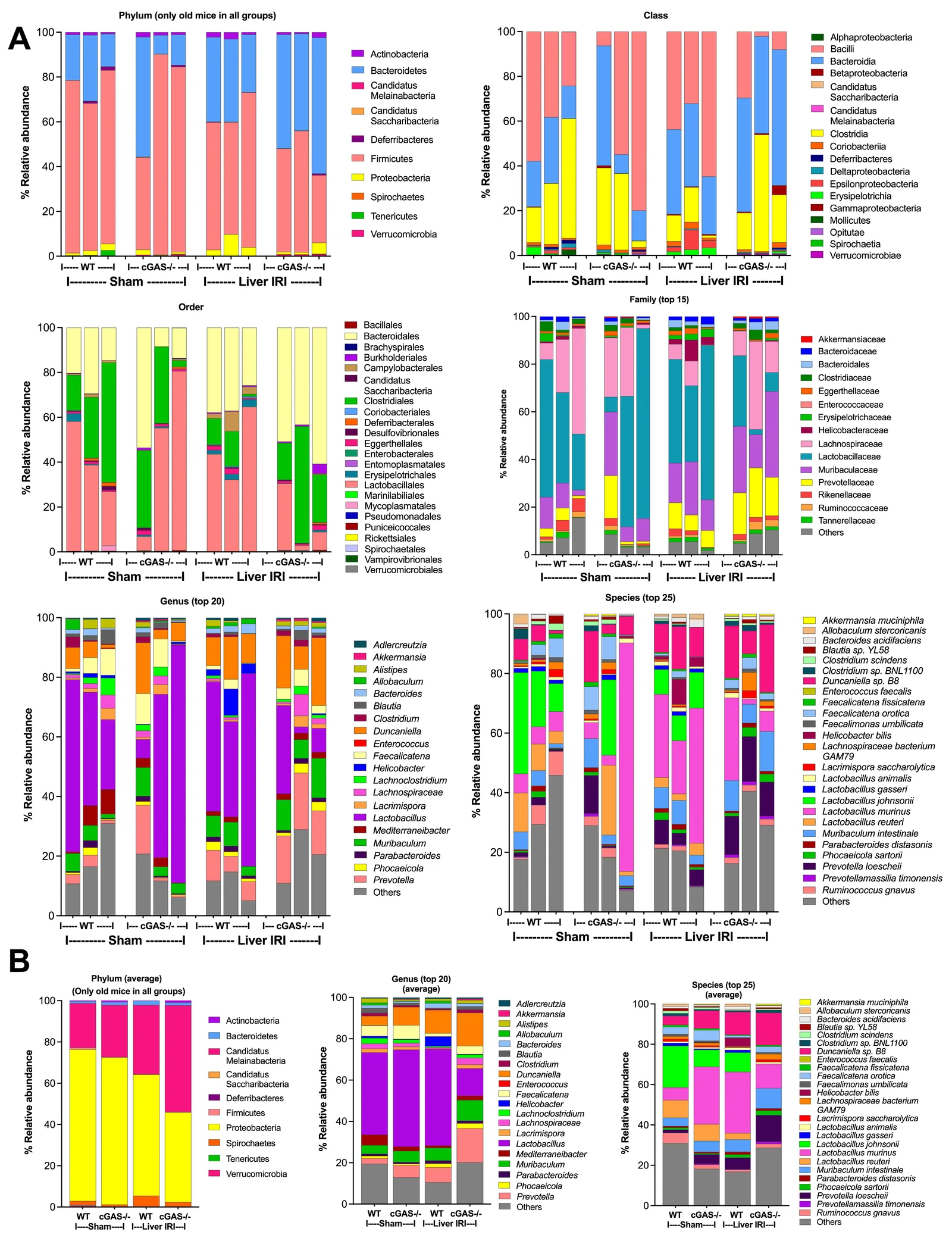
Fecal microbiome profiles of aged (56-week-old) wild-type (WT) and *cGAS*^-/-^ mice at 6 h post-hepatic ischemia–reperfusion injury (IRI) or sham surgery, illustrating (**A**) relative bacterial abundance across multiple taxonomic levels (phylum, class, order, family, genus, and species) and (**B**) average abundance specifically at the phylum, genus, and species levels. *n* = 3/group.

**Figure 6:**
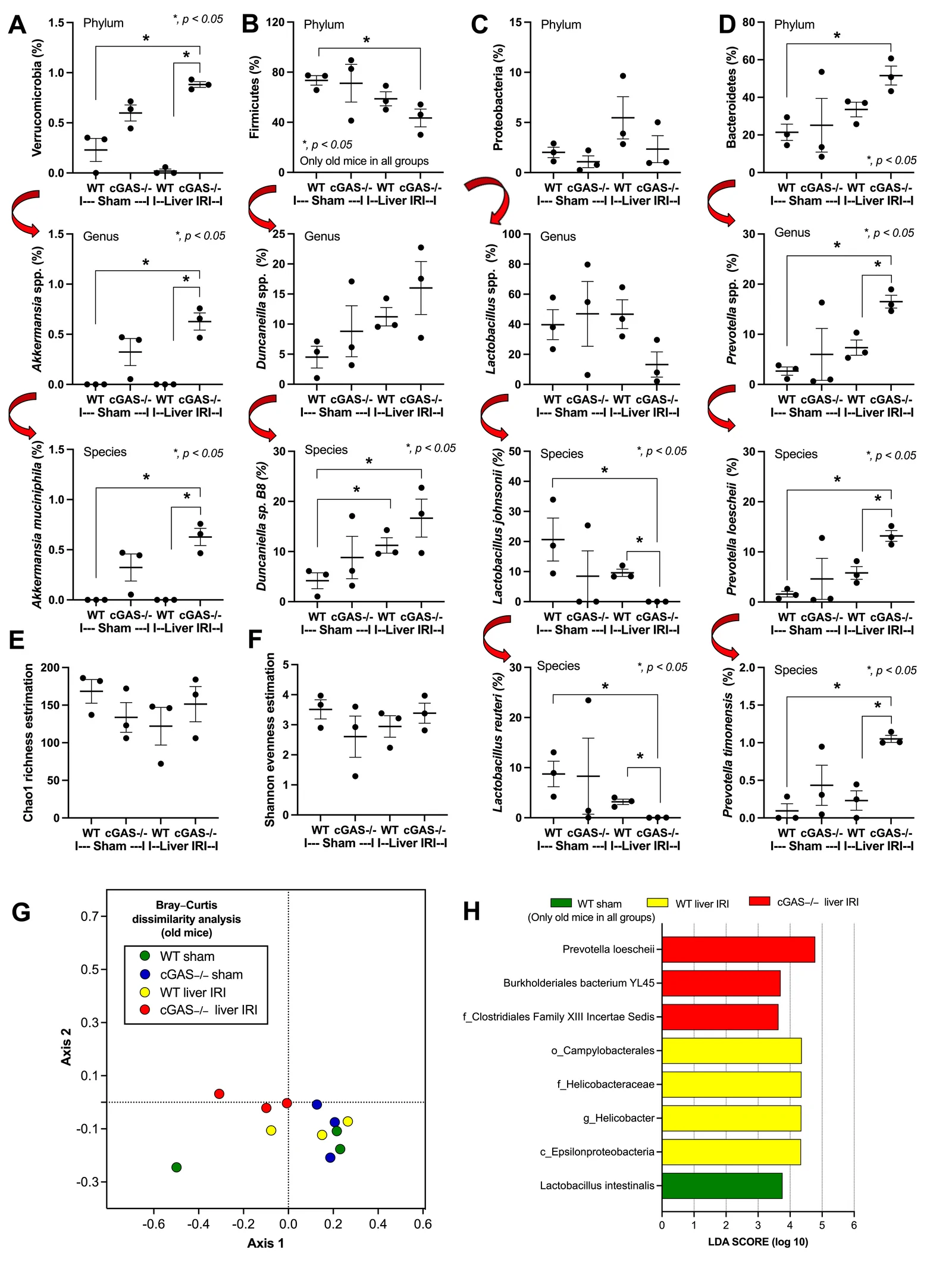
Fecal microbiome profiles of aged (56-week-old) wild-type (WT) and *cGAS*^-/-^ mice at 6 h post−hepatic ischemia–reperfusion injury (IRI) or sham surgery. The panels illustrate (**A**–**D**) relative bacterial abundance at the phylum, genus, and species levels; (**E**,**F**) alpha diversity assessed by Chao-1 and Shannon indices; (**G**) beta diversity based on Bray–Curtis dissimilarity; and (**H**) linear discriminant analysis effect size (LEfSe) highlighting differentially abundant taxa specific to each group (*n* = 3 per group). * *p* < 0.05.

Because of the possible correlation between gut barrier defect (leaky gut) and fecal microbiome alteration [38,39]. Fecal microbiome analysis of old mice at 6 h post-liver IRI (the time point positive for leaky gut; Figure 1O) was evaluated (Figure 5A,B). Although microbiota parameters did not differ between WT and cGAS^-/-^ feces after sham surgery (Figure 6A–D), a few differences emerged after liver IRI. For example, Verrucomicrobia (identified as Akkermansia muciniphila) and *Prevotella* spp. (identified as *P. loescheii* and *P. timonensis*), and lower *Lactobacillus johnsonii* and *L. reuteri* (no difference in the Lactobacillus at the species level) in *cGAS*^-/-^ than WT mice, without a difference in alpha diversity (Chao-1 and Shannon indexes) (Figure 6A–F). The beta diversity (the species diversity between groups), as indicated by the non-metric multidimensional scaling (NMDS) based on the Bray–Curtis (the simplified presentation of the difference between groups through the distances from the x and y axes), indicated a close proximity within *cGAS*^-/-^ liver IRI without the grouping of other groups (Figure 6G). The Linear Discriminant Analysis effect size (LEfSe; the representative bacteria of the group), calculated by Linear Discriminant Analysis (LDA), indicated a few differences (Figure 6H). As such, the representative bacteria in *cGAS*^-/-^ liver IRI were Prevotella, Burkholderia, and Clostridium; in WT liver IRI, Campylobacter, Helicobacter, and Proteobacteria; and in WT sham, *L. intestinalis* (Figure 6H). Although the crosstalk between liver injury and gut microbiome alteration was possible [40], the number of samples here was too few for a solid conclusion.

### 3.3. Macrophages and Hepatocytes, a Mechanistic Exploration

**Figure 7:**
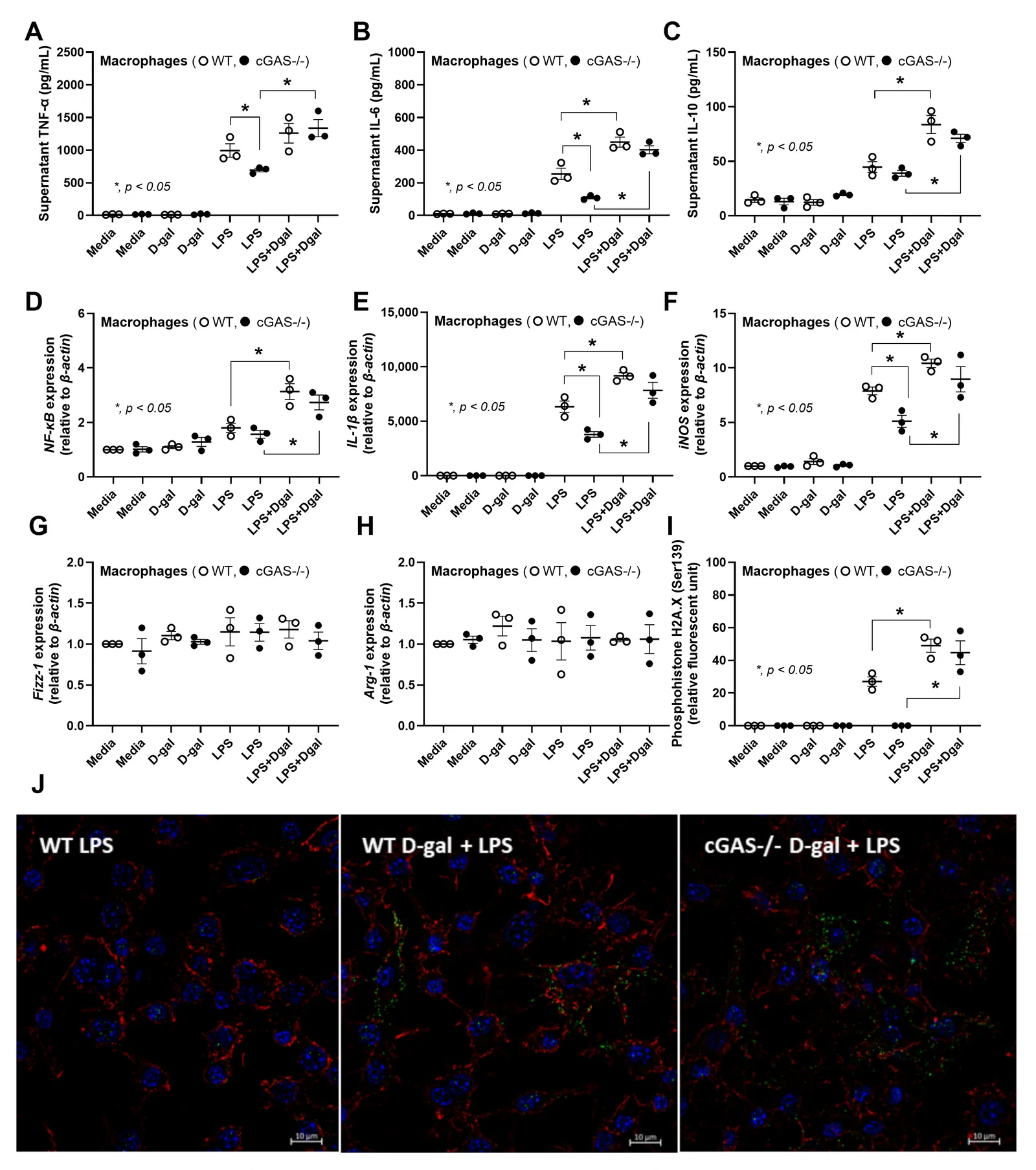
Phenotypic and inflammatory profiles of bone marrow-derived macrophages (BMDMs) from wild-type (WT) and cGAS^-/-^ mice at 24 h following stimulation with D-galactose (D-gal), with or without lipopolysaccharide (LPS), compared to media control. The panels illustrate (**A**–**C**) supernatant levels of the cytokines TNF-α, IL-6, and IL-10; (**D**) expression of the transcription factor NF-κB; (**E**,**F**) expression of M1 pro-inflammatory markers (IL-1β and inducible nitric oxide synthase [iNOS]); (**G**,**H**) expression of M2 anti-inflammatory markers (Fizz-1 and arginase 1 [Arg-1]); and (**I**,**J**) evaluation of DNA damage via phosphohistone H2A.X staining, accompanied by representative immunofluorescence images (positive signals shown in green). Data are representative of three independent experiments. * *p* < 0.05.

Given the well-established impact of aging-induced oxidative stress on macrophages [41] and the marked elevation of MDA and macrophage infiltration in the livers of aged *cGAS*^-/-^ mice post-IRI (Figure 1D,J), oxidative stress is hypothesized to drive this exacerbated aging-associated hepatic injury. Although D-galactose (D-gal) induces oxidative stress rather than true biological aging [42], its oxidation by galactose oxidase yields high levels of hydrogen peroxide and free radicals [43]. Therefore, D-gal was employed as an in vitro pilot model for stressed macrophages. Subsequently, macrophages were incubated with D-gal in the presence or absence of lipopolysaccharide (LPS), a representative activator. Without D-gal, *cGAS*^-/-^ macrophages showed reduced inflammatory responses, as indicated by supernatant TNF-α, IL-6, IL-1β, and iNOS expression, but not by IL-10, NF-κB, Fizz-1, Arg-1, or the DNA-break marker (phosphohistone H2A.X) (Figure 7A–I). With D-gal co-incubation, inflammatory responses and DNA break injury were elevated in both WT and cGAS^-/-^ macrophages (similar levels in WT and *cGAS*^-/-^ cells) (Figure 7A–J). Although *NF-κB* mRNA is not a readout of NF-κB activation, the levels reflect the transcriptional capacity, with slower changes than phosphorylation and proteasomal degradation [44]. The elevated *NF-κB* expression after LPS and D-gal co-incubation partly indicates the increased capacity of NF-κB to undergo nuclear translocation and initiate pro-inflammatory gene transcription [45]. Although the pro-inflammatory response of *cGAS*^-/-^ macrophages did not exceed that of wild-type (WT) cells following LPS and D-gal co-incubation, the enhanced activation observed in LPS/D-gal-treated macrophages compared to those treated with LPS alone highlights the impact of oxidative stress on macrophage function.

**Figure 8:**
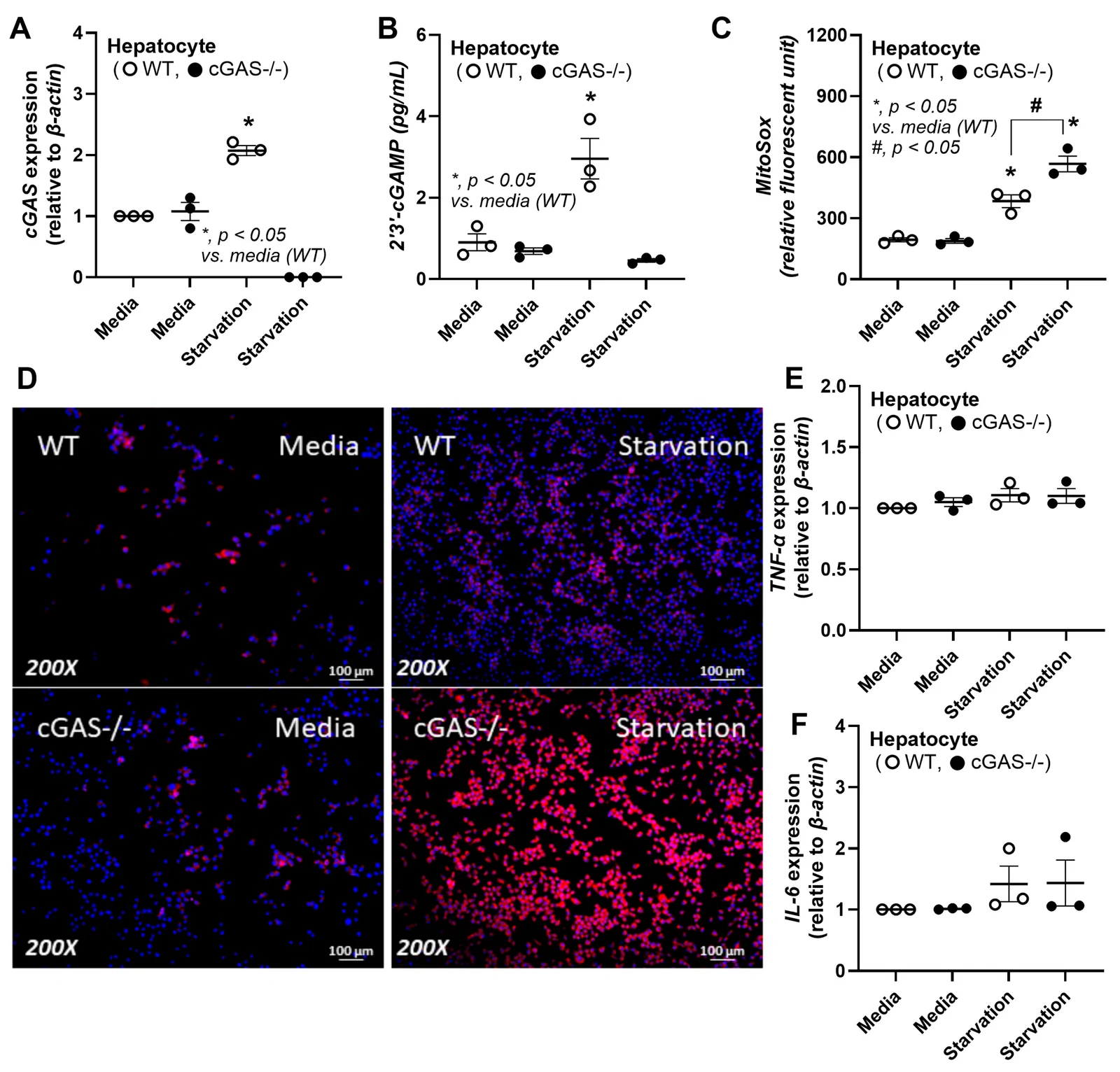
In vitro responses of hepatocytes from wild-type (WT) and cGAS^-/-^ mice at 24 h following a starvation protocol or media control. The panels illustrate (**A**) *cGAS* expression; (**B**) supernatant levels of 2′3′-cGAMP (a second messenger of cGAS); (**C**,**D**) mitochondrial oxidative stress evaluated by MitoSOX staining, along with representative fluorescence images; and (**E**,**F**) expression of the cytokine genes TNF-α and IL-6. Data are representative of three independent experiments. * *p* < 0.05 vs. media in WT; # *p* < 0.05.

In addition to macrophage accumulation, direct injury to hepatocytes [15] likely contributes to ischemia-induced liver damage. Rather than employing a standard in vitro hypoxia-reoxygenation procedure [46], a cell starvation protocol was utilized as a pilot model. The activation of the cGAS pathway during cellular starvation was confirmed by the expression of cGAS and the release of its second messenger, 2′3′-cGAMP, into the supernatant of WT hepatocytes (Figure 8A,B). Consistent with the known effects of *cGAS* deficiency [47], starvation-induced mitochondrial injury and subsequent oxidative stress were significantly exacerbated in *cGAS*^-/-^ hepatocytes compared to WT cells (Figure 8C,D), while cytokine gene expression remained unaltered (Figure 8E). Collectively, these findings suggest that the absence of cGAS during hepatocyte starvation induces severe mitochondrial dysfunction, which may account for the exacerbated hepatocellular death and macrophage abundance observed in vivo in *cGAS*^-/-^ mice following liver IRI (Figure 1I,J).

## 4. Discussion

Although loss of cGAS (a receptor for cytosolic DNA) might reduce macrophage inflammatory responses to liver IRI, hepatic cell death in aged *cGAS*^-/-^ mice was more prominent than in other groups, possibly due to aging-induced oxidative stress.

### 4.1. More Severe Liver Damage in Aged cGAS^-/-^ Mice, a Vulnerability of cGAS^-/-^ Hepatocytes to Stress

The cyclic GMP-AMP synthase (cGAS) is a DNA sensor floating in the cell cytosol, recognizing abnormal DNA from organisms, mitochondria, and host nuclei before binding to another cGAS (dimerization) [48], allowing it to use ATP and GTP to develop into 2′3′-cGAMP [49]. The highest level of liver damage in aged *cGAS*^-/-^ mice, with no difference between young *cGAS*^-/-^ and WT mice, implies that *cGAS*^-/-^ hepatocytes are more vulnerable to stress or injury in aged mice. Different from other cells, hepatocytes lack STING expression due to the high abundance of mitochondria and endoplasmic reticulum, with prominent metabolic stress that is prone to minor mitochondrial DNA leakage [50]. The non-STING expression in hepatocytes is a natural protection to avoid unnecessary chronic inflammation from regular metabolism while taking a risk of viral infection [51]. Without STING, cGAS in hepatocytes exerts a non-canonical, protective role to initiate autophagy (a process used to clear damaged organelles and metabolic waste) [52].

In this context, elevated *cGAS* expression and supernatant cGAMP levels after cell starvation in wild-type (WT) hepatocytes support cGAS involvement in hepatic stress (Figure 8A,B). Furthermore, the exacerbated mitochondrial oxidative stress (MitoSOX) observed in *cGAS*^-/-^ hepatocytes compared to WT cells suggests that *cGAS*-deficient cells are more vulnerable to injury. This in vitro susceptibility aligns with the extensive apoptosis observed in vivo within the livers of aged (56-week-old) *cGAS*^-/-^ mice following injury. Conversely, young *cGAS*^-/-^ mice showed a trend toward more severe ischemia-induced liver damage than their WT counterparts, but this difference did not reach statistical significance.

Despite exacerbated starvation-induced injury in *cGAS*^-/-^ hepatocytes in vitro, young *cGAS*^-/-^ and wild-type (WT) mice demonstrated comparable liver damage after ischemia–reperfusion injury (IRI), underscoring the critical influence of aging. Indeed, age-related factors, such as accumulated oxidative stress, vascular alterations, and impaired regenerative capacity, likely increase hepatic susceptibility to injury [3]. This age-associated vulnerability may drive the exacerbated hepatocellular death and prominent macrophage accumulation observed in aged (56-week-old) *cGAS*^-/-^ mice post-IRI (Figure 1J). However, the severity of ischemia-induced liver damage is likely dictated not only by intrinsic hepatocyte vulnerability but also by macrophage-mediated responses. Unlike hepatocytes, which lack STING expression, the cGAS-STING axis is robustly active in macrophages. Consequently, the loss of cGAS signaling in these immune cells results in an attenuated inflammatory response compared to wild-type (WT) macrophages [51].

In macrophages, co-stimulation with LPS and D-gal enhanced the pro-inflammatory response of *cGAS*^-/-^ cells, restoring it to wild-type (WT) levels. Although the effects of D-gal may stem from both oxidative stress induction and osmotic injury [51] (as osmotic controls such as mannitol or L-glucose were not included in our experiments), the combination of LPS and D-gal induced a markedly stronger macrophage activation than LPS alone, highlighting the substantial modulatory impact of D-gal. While the exact relative contributions of hepatocytes and macrophages to the observed *cGAS*^-/-^ liver damage remain to be fully elucidated, the extensive hepatic injury and prominent macrophage abundance in aged (56-week-old) *cGAS*^-/-^ mice likely drive the elevated serum IL-6 and impaired gut permeability at 6 h post-injury. Consequently, the severe damage in aged *cGAS*^-/-^ mice following liver IRI may primarily result from the heightened susceptibility of *cGAS*-deficient hepatocytes to injury, with the extensive cellular damage further amplifying cell death-induced inflammation.

In a previous study, male 8–12-week-old *cGAS*^-/-^ mice in the C57BL/6J background (Jackson Laboratory, Bar Harbor, ME, USA) with 60 min of 70% partial liver IRI demonstrated more severe liver injury than WT (C57BL/6J), possibly due to abnormal autophagy [15]. Here, we demonstrated only a tendency toward more severe liver injury in female 8-week-old *cGAS*^-/-^ mice than in WT (AST and ALT) (Figure 1F,G), with significantly higher caspase-3 and cell-free DNA levels than in WT (Figure 1I,K). This discrepancy may reflect gender differences or the mismatch between the *cGAS*^-/-^ background (C57BL/6J) and the WT background (C57BL/6NJcl) in this study. Although some underlying mechanisms remain to be fully elucidated, our findings underscore the profound impact of aging on inflammatory conditions. Crucially, these results suggest that conclusions drawn exclusively from young murine models may not accurately reflect the pathophysiology in aged subjects. Future studies that specifically address age-related variables in this context are warranted.

### 4.2. The Possible Differences in Fecal Microbiota Between Aged cGAS^-/-^ and WT Mice with Liver Injury, Another Interesting Factor

The liver injury-induced gut dysbiosis through impaired motility, disrupted barrier, and malabsorption [53,54], similar to stress- and exercise-induced dysbiosis [55,56], is established. Here, only the liver damage in aged *cGAS*^-/-^ liver IRI was severe enough to transiently elevate serum IL-6 and FITC-dextran (leaky gut) at 6 h post-liver IRI. However, no microbiome differences were observed between sham groups (WT and *cGAS*^-/-^), indicating a limited impact of cGAS on the fecal microbiome under normal conditions. After liver IRI, the *cGAS*^-/-^ microbiome was different from WT, including elevated *Prevotella* spp. (Bacteroides, Gram-negative anaerobes with possible harmful effects in some situations) [57,58,59,60], increased *Akkermansia* spp. (the Verrucomicrobia phylum with possible benefits and harmful effects) [61], and reduced *Lactobacillus* spp. (the Firmicutes phylum with well-known probiotic effects) [62]. While *Prevotella* spp. break down high-fiber diets and nourish enterocytes, their overgrowth in the gut is associated with chronic inflammation [63]. *Akkermansia muciniphila* is a Gram-negative bacterium that degrades mucus (mucin) as its only energy source, which might stimulate more mucin production or reduce the gut barrier [64]. Meanwhile, reduced *Lactobacillus* spp. in the gut may not benefit the host. However, the limited sample size in our microbiome analysis precludes definitive conclusions. Future studies that comprehensively explore gut microbiota alterations in this context are warranted.

### 4.3. Clinical Translation, Limitations and Future Directions

For clinical translation, several small molecules for cGAS blockage are currently being studied to block abnormal innate immune responses and inflammation caused by cytosolic dsDNA in several autoimmune diseases [65]. Consequently, clinicians must consider the detrimental effects of hepatocellular cGAS inhibition when using systemic cGAS blockade in clinical settings. Therefore, when administering cGAS inhibitors, prioritize rigorous monitoring of liver function and avoid concurrent hepatotoxic conditions.

Several limitations of this study should be noted. First, the in vivo experiments involved certain technical constraints, notably the use of different mouse substrains (*cGAS*^-/-^ on a C57BL/6J background versus WT on a C57BL/6NJcl background) without littermate controls. Additional limitations include the absence of co-housing protocols to normalize the gut microbiota, the lack of sham-operated controls in the time-course experiments, the exclusive use of female mice, and the omission of direct oxidative stress manipulation assays. As reported in other models [66], these substrain variations may partially account for the divergent results observed in our study. Consequently, the phenotypic differences between WT and *cGAS*^-/-^ mice may be partly attributable to their distinct genetic backgrounds. Future studies need to rigorously control for these genetic variables. Furthermore, the constitutive *cGAS* knockout model used here ablates the gene globally across all cell types, including both hepatocytes and macrophages. Thus, subsequent mechanistic investigations utilizing conditional knockout models are highly warranted to delineate cell-specific roles. For example, utilizing conditional knockout strategies, such as crossing *cGAS*-flox mice with *Alb*-Cre or *LysM*-Cre lines, would enable the targeted deletion of *cGAS* specifically in hepatocytes or myeloid cells, respectively [67,68]. Combining these genetic models with the evaluation of downstream hepatic signaling (cGAMP, p-TBK1/p-IRF3, and IFN-β/ISGs) [69], macrophage depletion via liposomal clodronate [70], and antioxidant interventions (such as NAC in aged mice) [71] would provide more conclusive mechanistic data. Furthermore, the present study did not delineate the relative cell-intrinsic contributions of hepatocytes versus macrophages to the observed *cGAS*^-/-^ phenotype. Future studies that isolate and ex vivo characterize these primary cells would clarify their distinct roles and yield more robust mechanistic conclusions. Despite the inconclusive results, our data suggested that aging affects *cGAS*^-/-^ mice. More rigorous studies, especially in male mice, are needed.

Second, fecal microbiome analysis was performed as a pilot experiment only at 6 h post-liver IRI in aged mice using only 3 mice per group. Evaluating the fecal microbiome at additional time points and in young cohorts, utilizing an experimental design specifically tailored to investigate the gut-liver axis, would likely provide deeper mechanistic insights.

Third, the in vitro experiments had several technical limitations, including the use of glucose starvation and D-galactose. The hypoxia/reoxygenation model in primary hepatocytes may better reflect in vivo conditions. In addition, D-galactose-induced oxidative stress differs from that in chronologically aged cells. The separation of Kupffer cells from aged mice, including Clec4f/Tim4 (the resident Kupffer cells) [72] or CCR2/Ly6C (recruited monocytic lineage) [73], might be more appropriate. Fourth, the impacts of aging on inflammation might involve not only oxidative stress accumulation but also other factors (hormones, atherosclerosis, and organ capacities) [3]. Exploring other aging-related factors is also interesting.

## 5. Conclusions

In conclusion, the combination of aging and *cGAS* deficiency, rather than either factor alone, appears crucial for the severe liver damage observed following ischemia–reperfusion injury (IRI). In young *cGAS*^-/-^ mice, the heightened susceptibility of hepatocytes to starvation-induced injury may be offset by reduced inflammatory responses in *cGAS*^-/-^ macrophages, resulting in liver damage comparable to WT mice. However, in aged mice, age-related factors further exacerbate the injury in *cGAS*-deficient hepatocytes. Therefore, aging plays a critical role in enhancing hepatic vulnerability in *cGAS*^-/-^ mice during liver IRI.

## Figures and Tables

**Table 1:** Primers used in the study.

Name	Forward Primer	Reverse Primer
Nuclear factor-kappa B (*NF-κB*)	5′-CTTCCTCAGCCATGGTACCTCT-3′	5′-CAAGTCTTCATCAGCATCAAACTG-3′
Interleukin 1-beta (*IL-1β*)	5′-CCACAGACCTTCCAGGAGAATG-3′	5′-GTGCAGTTCAGTGATCGTACAGG-3′
Inducible nitric oxide synthase (*NOS-2*)	5′-CAGCGGGATGACTTTCCAAG-3′	5′-AGGCAAGATTTGGACCTGCA-3′
Arginase-1 (*Arg-1*)	5′-TGGACAGACTAGGAATTGGCA-3′	5′-CCAGTCCGTCAACATCAAAACT-3′
Resistin-like alpha (*Fizz-1*)	5′-GCCAGGTCCTGGAACCTTTC-3′	5′-GGAGCAGGGAGATGCAGATGA-3′
Cyclic GMP–AMP synthase (*cGAS*)	5′-ATGTGAAGATTTCGCTCCTAATGA-3′	5′-GAAATGACTCAGCGGATTTCCT-3′
Tumor necrosis factor alpha *(TNF-α)*	5′-CTCACACTCAGATCATCTTCTC-3′	5′-AGATCCATGCCGTTGGCCAG-3′
Interleukin 6 *(IL-6)*	5′-TACCACTTCACAAGTCGGAGGC-3′	5′-CTGCAAGTGCATCATCGTTGTTC-3′
*β-actin*	5′-CCTGGCACCCAGCACAAT-3′	5′-GCCGATCCACACGGAGTACT-3′

## Data Availability

The data are contained within the article.

## Supplementary Materials

The following supporting information can be downloaded at https://www.mdpi.com/article/10.3390/biology15212021/s1. Table S1. The interaction *p*-values from the two-way ANOVA analyses.

## Abbreviations

The following abbreviations are used in this manuscript:

cfDNA	cell-free DNA
cGAS	cyclic GMP-AMP synthase
cGAMP	cyclic guanosine monophosphate–adenosine monophosphate
Liver IRI	Liver ischemic reperfusion injury
LPS	Lipopolysaccharide
STING	Stimulator of interferon genes
ROS	Reactive oxygen species
p-TBK1	Phospho-TANK-binding kinase 1
p-IRF3	Phospho-interferon regulatory factor 3
IFN-β	Interferon beta
ISGs	Interferon-stimulated genes
