# Supplementary material for: Prominent Hepatic Injury in 56-Week-Old Female Mice with Cyclic Guanosine Monophosphate–Adenosine Monophosphate Synthase (cGAS) Deficient
Source: Biology (Basel). 2026 Nov 6;15(21):2021. doi: 10.3390/biology15212021 (PMC13762361; doi:10.3390/biology15212021)
Supplement: Supplementary file 1 [file biology-15-02021-s001.zip › biology-4615609-supplementary.pdf]

Table S1. The interaction *p-values* from the two-way ANOVA analyses

Age × Genotype interaction

**Outcomes**	**Timepoint**	***p-value***
Serum ALT	6 h	0.0079
	24 h	0.0010
Serum AST	6 h	0.0152
	24 h	0.0462
Histology injury (Suzuki's score)	6 h	0.0359
	24 h	0.0424
Apoptosis score	6 h	0.0467
	24 h	0.0300
Macrophage accumulation	6 h	0.0091
	24 h	0.0487
